# A Behavioral Genetic Study of Intrapersonal and Interpersonal Dimensions of Narcissism

**DOI:** 10.1371/journal.pone.0093403

**Published:** 2014-04-02

**Authors:** Yu L. L. Luo, Huajian Cai, Hairong Song

**Affiliations:** 1 Key Laboratory of Behavioral Science, Institute of Psychology, Chinese Academy of Sciences, Beijing, China; 2 Department of Psychology, University of Oklahoma, Norman, Oklahoma, United States of America; University of Granada, Spain

## Abstract

Narcissism, characterized by grandiose self-image and entitled feelings to others, has been increasingly prevalent in the past decades. This study examined genetic and environmental bases of two dimensions of narcissism: intrapersonal grandiosity and interpersonal entitlement. A total of 304 pairs of twins from Beijing, China completed the Narcissistic Grandiosity Scale and the Psychological Entitlement Scale. Both grandiosity (23%) and entitlement (35%) were found to be moderately heritable, while simultaneously showing considerable non-shared environmental influences. Moreover, the genetic and environmental influences on the two dimensions were mostly unique (92–93%), with few genetic and environmental effects in common (7–8%). The two dimensions of narcissism, intrapersonal grandiosity and interpersonal entitlement, are heritable and largely independent of each other in terms of their genetic and environmental sources. These findings extend our understanding of the heritability of narcissism on the one hand. On the other hand, the study demonstrates the rationale for distinguishing between intrapersonal and interpersonal dimensions of narcissism, and possibly personality in general as well.

## Introduction

The cover story for the May 20, 2013 issue of *Time* magazine entitled “*Millennials: The Me Me Me Generation*” depicted so-called *millennials* - the youth generation spanning the 1980s to 2000 - as typical narcissists: “*Millennials are lazy, entitled narcissists, who still live with their parents”* [1]. Indeed, recent research has extensively documented the increasing prevalence of narcissism [2]–[5]. In this study, what we are interested in are the genetic bases of narcissism. Research has demonstrated that narcissism in general is heritable [6]–[10]. We aim to further examine the genetic bases of its sub-dimensions, in particular, of the intrapersonal and interpersonal dimensions.

Narcissism refers to a kind of abundant self-love, characterized by a series of characteristics including self-desire for admiration, fantasies of superiority, hypersensitivity to criticism, exploitation of people, and lack of empathy for others [11]. In social-personality psychology, narcissism primarily has been measured by the Narcissistic Personality Inventory (NPI) [12]–[14]. A number of studies utilizing factor analyses have been performed on the NPI. Early works revealed a four-factor structure: Leadership/Authority, Self-Absorption/Self-Admiration, Superiority/Arrogance, and Exploitiveness/Entitlement [12], [13], or a seven-factor structure: Authority, Exhibitionism, Superiority, Entitlement, Exploitiveness, Vanity, And Self-Sufficiency [15]. More recently, some researchers have proposed more parsimonious models, such as two-factor model and three-factor model [16]–[18]. Although the number of underlying factors is inconclusive, no one disagrees that narcissism is a multi-factor construct.

Nevertheless, it is evident that characteristics of narcissism or factors underlying the NPI tend to fall into two clusters: one involving intrapersonal attributes such as superiority and self-sufficiency, and the other involving interpersonal traits such as exploitiveness and entitlement. Indeed, high agency but low communion has been widely accepted as the core features of narcissists [19]–[22]. At one extreme, narcissists feel grandiose, superior, and powerful; at the other extreme, they also feel fully entitled, to the extent that they have appropriated the mindset that “others exist for me” [23]. Based on intrapersonal and interpersonal distinctions, researchers have actually suggested conceptualizing narcissism along the two dimensions of grandiosity and entitlement [24]. In this context, grandiosity refers to “*a grandiose sense of self-importance*” and denotes the core nature of intrapersonal features, whereas entitlement refers to “*an entitled, socially objectifying sense of the self in relation to others*” and represents the core nature of the interpersonal dimension of narcissism (p. 953, [24]). Empirical research has established the differential nature of intrapersonal and interpersonal dimensions of narcissism. For example, by using grandiosity and entitlement as representative features, research has demonstrated that the intrapersonal and interpersonal dimensions of narcissism manifest distinct patterns of associations with the five factors of personality [25], indexes of mental health such as life satisfaction, life orientation, depression, and self-esteem [24], various aspects of social cognition [26], early life experience [27], and diverse ways to enhance their self-image [24], [28].

In this study, what fascinate us are the genetic and environmental bases of the intrapersonal and interpersonal dimensions of narcissism. Research in the West has consistently showed that narcissism is heritable regardless its various standards of measurement [6]–[9]. The latest research indicated that narcissism also has a genetic base in the East [10]. Built on these studies, the purpose of our current study is twofold: first, we examine whether the intrapersonal and interpersonal dimensions of narcissism are heritable; second, given the distinctive nature of the intrapersonal and interpersonal features of narcissism, we examine whether these two distinct dimensions have differential genetic and environmental bases. To our knowledge, this is the first study devoted to the heritability of the specific dimensions of narcissism.

We focused on grandiosity and entitlement, which have been demonstrated to be representative of the intrapersonal and interpersonal dimensions of narcissism, respectively [24]. Similar to the previous study [24], we used the Narcissistic Grandiosity Scale [24], [25] and the Psychological Entitlement Scale [29] as measures of grandiosity and entitlement, respectively. Based on prior twin studies on narcissism and studies on the distinction between grandiosity and entitlement, we formulated two hypotheses. First, we expected that the two dimensions of narcissism, grandiosity and entitlement would be heritable; meanwhile, we also expected to find considerable influence on the two dimensions from non-shared environments, i.e., environments unique to each sibling (*Hypothesis* 1). Second, more important, we expected that the two dimensions of narcissism would reveal differential genetic and environmental bases (*Hypothesis* 2). To test these hypotheses, we first used two univariate genetic models to examine the heritability of grandiosity and entitlement separately, and then utilized two bivariate genetic models to analyze genetic as well as environmental influences on grandiosity and entitlement simultaneously.

## Methods

### Ethics Statement

The Ethics Committee of the Institute of Psychology, Chinese Academy of Sciences provided approval for the study. Additionally, we obtained written informed consent from all participants and their parents prior to commencing the test.

### Participants

A sample of 304 twin pairs from the Beijing Twin Study (BeTwiSt) participated in the current study. Twins in the BeTwiSt are socio-demographically representative of their peers in Beijing in general [30]. The participants in the present study ranged in age from 15 to 27 years (*M* = 18.29, *SD* = 1.96; 44.1% male). Of them, 152 pairs are monozygotic (MZ) whereas 152 pairs are dizygotic (DZ; 94 same-sex, 58 opposite-sex). To determine zygosity, DNA testing, with a classification accuracy approaching 100%, was used for 95% of the twin pairs; for the remaining pairs, zygosity was established by a combination of parent-reports and children’s self-reports about co-twin physical similarity and frequency of confusion, which had a predictive accuracy of 90.6% [31]. One member of a DZ twin pair was ultimately jettisoned because of missing data.

### Measures

All participants completed measures of grandiosity and entitlement, i.e., the Narcissistic Grandiosity Scale [24], [25] and the Psychological Entitlement Scale [29] along with other irrelevant measures, on personal computers in quiet, private rooms. All measures were translated into Chinese. Translation and back-translation procedures were employed to ensure the equivalence across languages.

The *Narcissistic Grandiosity Scale* consists of 16 trait adjectives (i.e., *perfect, omnipotent, powerful, extraordinary, outstanding, brave, unique, powerful, admirable, prestigious, honorable, excellent, respectable, talented, advanced, enviable, and vigorous*) which clearly reflect a grandiose sense of self-importance [24], [25]. Participants were asked to rate the extent to which each generally describes them on a 7-point Likert scale ranging from 1 (*not at all*) to 7 (*extremely*). The internal consistency was high (α = 0.95). For each respondent, the average rating across the 16 items formed the final score, with a larger value indicating a stronger sense of grandiosity.

The *Psychological Entitlement Scale* contains nine statements such as “I honestly feel I’m just more deserving than others” and “I feel entitled to more of everything” [29]. Participants indicated their agreement with each statement on a 7-point Likert scale (1 = *strongly disagree*, 7 = *strongly agree*). Internal consistency was desirable (α = 0.84). As in the above test, the average score was obtained for each participant, with a high score indicating a high endorsement of entitlement.

### Genetic Analyses

Standard quantitative genetic modeling decomposes phenotypic variance within a trait or covariance between traits into three components: additive genetic effect (A); shared environmental effect (C); and non-shared environmental effect (E; also includes measurement error). The term “heritability” denotes the proportion of the variance/covariance explained by the genetic effect. While shared environment contributes to the similarity of twins growing up in the same family, non-shared environment is unique to each individual.

First, to examine the heritability of grandiosity and entitlement, we used *univariate models* implemented in the OpenMx library [32] within the R statistical computing environment [33]. The models partitioned variances of grandiosity and entitlement, respectively, into ACE components.

**Figure 1:**
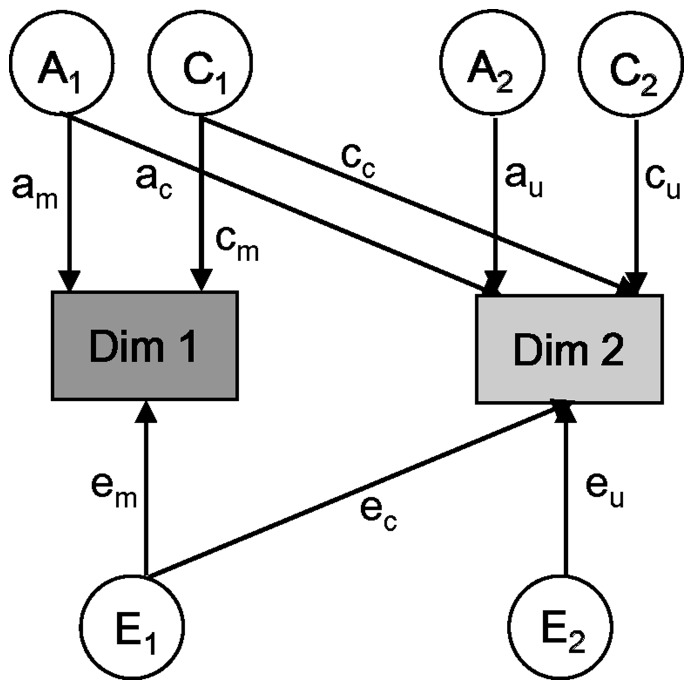
Path diagram illustrating the bivariate genetic model. *Note.* Dim = dimension. Dim 1/2 represents either one of the two narcissistic dimensions, grandiosity and entitlement. Measured variables are depicted in rectangles. Latent factors A (additive genetic factor), C (shared environmental factor), and E (non-shared environmental factor) are presented in circles. c = common; u = unique; m = main (main influence on the first measured variable from A_1_/E_1_).

To further assess the relationship between genetic as well as environmental influences on grandiosity and entitlement, we used the bivariate *Cholesky decomposition* model implemented in the OpenMx library. The Cholesky decomposition is similar to hierarchical regression analyses in non-genetic studies, through which the independent contribution of predictors entered later is assessed after controlling for the predictors entered first [34]. The bivariate model parameterized the variances for and the covariance between grandiosity and entitlement into “common factors” (A_1_, C_1_, E_1_) influencing both measures, and into “specific factors” (A_2_, C_2_, E_2_) unique to the variable entered later in the model (Figure 1). To be noted, the “common factors” also include influences specific to the first variable in the model [34]. Hence, we entered grandiosity before entitlement in one model to test common and specific genetic as well as environmental influences on entitlement; then we entered entitlement before grandiosity in a second model to test those influences on grandiosity. Notably, the two models operate in the same way except that the order of entering the variables is reversed. From the model paths, we can estimate genetic, shared, and non-shared environmental effects on grandiosity and entitlement in addition to the correlations between those effects (i.e., genetic, shared, and non-shared environmental correlations).

To test the fit of the genetic models, the full ACE model was fitted first, followed by the three nested sub-models: AE model (with C removed), CE model (with A removed), and E model (with both A and C removed). The chi-square difference test was used to compare each sub-model with the full model with the aim of choosing the best-fit model. A significant chi-square difference (Δχ^2^) indicates that the nested model fits significantly more poorly than the full model, leading to the decision to retain the full model. Otherwise, in terms of parsimony [35], [36], the nested model (with fewer parameters than the full model) is considered a better-fit model. The Bayesian Information Criterion (BIC; [37]) was used to compare non-nested models, i.e., the AE, CE, and E models. The lower the BIC value, the better the fit.

## Results

### Descriptive Statistics

Consistent with previous studies [24], [25], grandiosity (*M* = 4.16, *SD* = 1.07) and entitlement (*M* = 2.87, *SD* = 0.98) were moderately correlated with each other (*r* = .28, *p*<0.001). Multiple regressions, with the scores of grandiosity or entitlement as criteria, showed that neither sex nor age could significantly predict any narcissistic measure (*p*s ≥0.1), with the exception of a modest effect of sex on grandiosity (*β* = −0.16, *p*<0.001). In line with earlier finding [24], this difference showed that males viewed themselves slightly more grandiose than females did.

Nevertheless, because twins are perfectly correlated for age and same-sex twins are perfectly correlated for sex, variation associated with age or sex may inflate the correlation between twins. Following standard procedure, all measures were corrected for age and sex effects using a regression procedure and standardized residuals were saved for genetic analyses [38]. For each measure, participants whose score were 3 SD beyond the mean value of the whole sample were excluded from genetic analyses, based on the standardized residuals, resulting in the exclusion of the data of one MZ twin pair on entitlement (see Table 1 for valid sample size). In the model fitting of each measure, all data available were included to increase statistic power even if the data of several twin pairs were not pairwised.

**Table 1:** Twin intraclass correlations (ICC) of grandiosity and entitlement.

Measure	Grandiosity_2	Entitlement_2	N
MZ			
Grandiosity_1	.58 (.42–.69)	−.05 (−.45–.24)	152
Entitlement_1	.25 (−.03–.46)	.51 (.33–.65)	151
DZ			
Grandiosity_1	.44 (.22–.59)	.22 (−.08–.43)	151
Entitlement_1	.01 (−.36–.28)	.27 (.00–.47)	151

*Note.* N = number of twin pairs with pairwised data. MZ = monozygotic twins; DZ = dizygotic twins; twin_1_ and twin_2_ are differentiated by the endings _1 and _2, respectively. 95% confidence intervals of ICC are in parentheses. The within-trait twin correlations are on the diagonal. The cross-trait twin correlations are above and below the diagonal.

### Univariate Analyses

We first examined the heritability of grandiosity and entitlement using univariate analyses. The twin intraclass correlations for both measures are shown in Table 1. The univariate model fit statistics and estimates of genetic and environmental effects are presented in Table 2.

**Table 2:** Univariate genetic model-fitting: model fit and parameter estimates (95% confidence intervals in parentheses).

Measure	Model	−2LL	*df*	Δχ^2^	Δ*df*	*p*	BIC	a^2^	c^2^	e^2^
Grandiosity	ACE	1679.92	603					0.23 (.00–51)	0.17 (.00–.42)	0.60 (.49–.74)
	AE	1681.03	604	1.11	1	0.29	−4.61	0.42 (.30–.53)		0.58 (.47–.70)
	CE	1681.29	604	1.37	1	0.24	−4.35		0.34 (.24–.44)	0.66 (.56–.76)
	E	1719.49	605	39.57	2	0.00	28.13			1.00 (1.00–1.00)
Entitlement	ACE	1683.83	602					0.35 (.01–47)	0.00 (.00–.25)	0.66 (.53–.80)
	AE	1683.83	603	0.00	1	1.00	−5.72	0.35 (.21–.47)		0.66 (.53–.80)
	CE	1687.95	603	4.13	1	0.04	−1.59		0.24 (.13–.34)	0.76 (.66–.87)
	E	1705.90	604	22.07	2	0.00	10.64			1.00 (1.00–1.00)

*Note.* −2LL = twice the negative log-likelihood, the difference between −2LL of two models is subjected to chi-square (χ2) distribution. Two fit indices are reported: change in chi-square (Δχ2) and Bayesian Information Criterion (BIC). Δ*df* = change in degrees of freedom (*df*). a^2^, c^2^, and e^2^ are proportion of variance due to additive genetic (A), shared environmental (C) and non-shared environmental effect (E). E, CE, and AE models are nested within ACE; the best fitting model is underlined.

#### Grandiosity

On feelings of grandiosity, MZ twins were related to each other to a greater extent than DZ twins were (Fisher’s z-test, one-tailed, *z* = 1.64, *p* = 0.05, Table 1), indicating that grandiosity is heritable. We then used the univariate ACE model to fit the twins’ grandiosity scores (Table 2). Results showed modest genetic effect (23%) as well as shared environmental influence (17%) but substantial non-shared environmental contribution (60%). Next, we compared the ACE model with its nesting sub-models. Both AE and CE models were comparable to the ACE model, but the E model was significantly worse. Compared with CE model, the AE model had lower BIC value. Therefore, the AE model was more desirable [35], [36]. It is worth noting that the heritability estimate (42%) was higher for the AE model than that for the full ACE model, because besides genetic effect, the A in the AE model also included the limited amount of variance caused by shared environment as shown in the full ACE model.

#### Entitlement

MZ twins resembled each other more than DZ twins did on the endorsement of entitlement (Fisher’s z test, one-tailed, *z* = 2.46, *p* = 0.007, Table 1), suggesting that one’s genetic makeup exerts substantial influence on entitlement. As a result, we devised univariate model-fitting to estimate the genetic and environmental contributions to the individual differences in entitlement (Table 2). Based on the results from the ACE model, 35% of individual difference in entitlement was attributed to additive genetic factors and the other 66% explained by non-shared environment, with an estimate of zero for shared environment. (Due to rounding, the sum of ACE estimates may not be exactly 100%.) As for the nesting models, the AE model fitted the data no worse than the full ACE model, whereas the CE and E models significantly reduced the fitness. Following the parsimony principle [36], the AE model provided the best account of the variance in entitlement.

### Bivariate Analyses

**Figure 2:**
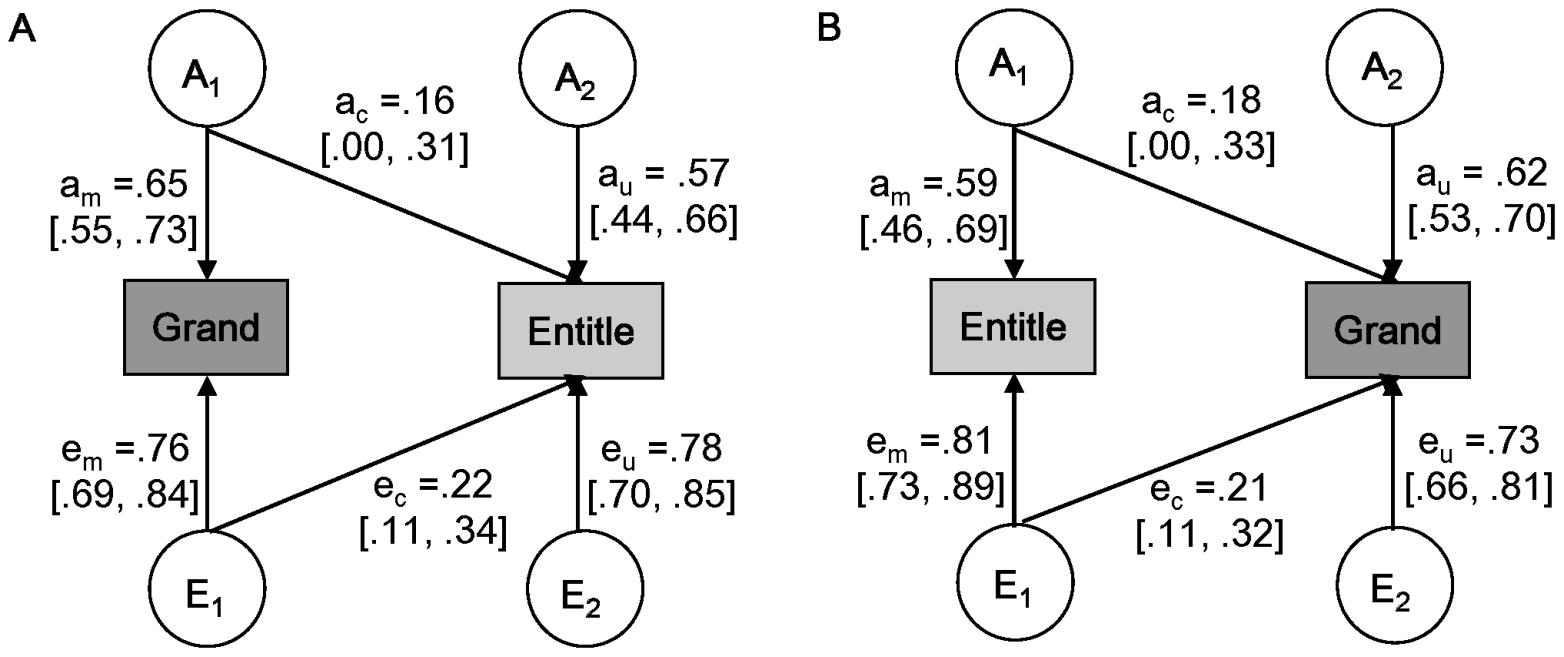
Bivariate genetic analysis of grandiosity and entitlement. *Note.* A) The best fitting bivariate model for influences from grandiosity to entitlement. B) The best-fitting bivariate model for influences from entitlement to grandiosity. Grand = Grandiosity; Entitle = Entitlement. Measured variables are depicted in rectangles. Latent factors A (additive genetic factor) and E (non-shared environmental factor) are presented in circles. c = common; u = unique; m = main (main influence on the first measured variable from A_1_/E_1_). Standardised (unsquared) path estimates and 95% confidence intervals are included. All the path estimates are obtained from the best fitting model, i.e., AE model.

Table 1 displays cross-twin cross-trait correlations for grandiosity and entitlement. Two bivariate models, as shown in Figure 1, were applied to analyze the genetic and environmental influences from grandiosity to entitlement and from entitlement to grandiosity, respectively. Consistent with the results from univariate modeling of grandiosity and entitlement, the AE model provided the best fit to the data for both models (Figure 2, Table 3). The AE model had the lowest BIC value and demonstrated a non-significant change in the chi-square compared to the full ACE model. Therefore, we adopted the AE model to estimate bivariate genetic and environmental influences. To note, the two chosen AE models fitted the data equally well, because they functioned in the same way, except the order of variables (i.e., grandiosity and entitlement), on the same data.

**Table 3:** Bivariate genetic model-fitting: model fit.

Model	−2LL	*df*	Δχ^2^	Δ*df*	*p*	BIC
Grandiosity → Entitlement (Figure 2.A)						
ACE	3315.51	1202				
AE	3317.28	1205	1.77	3	0.62	−15.39
CE	3320.59	1205	5.08	3	0.17	−12.07
E	3379.83	1208	64.32	6	0.00	30.02
Entitlement → Grandiosity (Figure 2.B)						
ACE	3315.51	1202				
AE	3317.28	1205	1.77	3	0.62	−15.39
CE	3320.59	1205	5.08	3	0.17	−12.07
E	3379.83	1208	64.32	6	0.00	30.02

*Note.* −2LL = twice the negative log-likelihood, the difference between −2LL of two models is subjected to chi-square (χ2) distribution. Two fit indices are reported: change in chi-square (Δχ2) and Bayesian Information Criterion (BIC). Δ*df* = change in degrees of freedom (*df*). A = additive genetic effects; C = shared environmental effects; E = non-shared environmental effects. E, CE, and AE models are nested within ACE model. The best fitting model is underlined.

As seen in Figure 2. A, the genetic effect on entitlement was composed of two parts. One part was caused by common genetic factors (a_c_^2^), which explained 7% (i.e., 0.16^2^/(0.16^2^+0.57^2^)) of the total genetic effect. The other was generated by unique genetic factors (a_u_^2^), which accounted for a much larger proportion of 93% (i.e., 0.57^2^/(0.16^2^+0.57^2^)). Similarly, the non-shared environmental effect also included two parts, with the unique part accounting for 93% (i.e., 0.78^2^/(0.22^2^+0.78^2^)) of the total effect and the common part accounting for the remaining 7% (i.e., 0.22^2^/(0.22^2^+0.78^2^)) (Figure 2. A). These results indicated that individual difference in entitlement originates from genetic and non-shared environmental sources mostly distinct from grandiosity, although genetic and non-shared environmental factors underlying grandiosity also influence entitlement to a minimal extent.

Likewise, genetic influence on grandiosity consisted of two parts (Figure 2.B): one due to the genetic effect shared with entitlement, and the other attributed to an effect unique to itself. The shared genetic effect accounted for 8% (i.e., 0.18^2^/(0.18^2^+0.62^2^)) of the genetic contribution, while the unique genetic effect explained 92% (i.e., 0.62^2^/(0.18^2^+0.62^2^)) of the genetic contribution. The non-shared environmental influence on grandiosity may also be broken down into two distinct sets (Figure 2.B). The majority being 92% (i.e., 0.73^2^/(0.21^2^+0.73^2^)) of the influence drew from environments unique to grandiosity. Environments common to grandiosity and entitlement accounted 8% (i.e., 0.21^2^/(0.21^2^+0.73^2^)) of the influence. Our findings suggested that the genetic and non-shared environmental foundation of grandiosity is largely different from that of entitlement, with only a slight overlap.

In addition, we obtained genetic and non-shared environmental correlations between grandiosity and entitlement in order to assess the relationship between their genetic and non-shared environmental factors. In keeping with the small genetic contribution from common factors, the genetic correlation between grandiosity and entitlement was 0.27 (i.e., a_c_/√(a_c_^2^+a_u_^2^) = 0.16/√(0.16^2^+0.57^2^) or 0.18/√ (0.18^2^+0.62^2^), 95% CI: .00–.50), confirming that only a small portion of genetic effects were held in common. It should be noted that the genetic correlation was not statistically significant because the lower bound of its 95% confidence interval was zero. The non-shared environmental correlation between grandiosity and entitlement was 0.28 (i.e., e_c_/√ (e_c_^2^+e_u_^2^) = 0.22/√(0.22^2^+0.78^2^) or 0.21/√(0.21^2^+0.73^2^), 95% CI:.14–.40), corroborating that environmental influences are largely unique to grandiosity and entitlement. Taken together, the genetic and environmental bases underlying grandiosity and entitlement are independent, demonstrating minor overlaps.

## Discussion

We are living in a narcissistic age. Narcissists attach superfluous importance and excellence to themselves at the same time they enjoy exploiting and manipulating others. In this study, we investigated the genetic bases of the intrapersonal and interpersonal dimensions of narcissism with grandiosity and entitlement as proxies. Corroborating our *hypothesis* 1, we found that both intrapersonal grandiosity and interpersonal entitlement are moderately heritable (the heritability is 23% and 35%, correspondingly), with considerable non-shared environmental influence but small even null shared environmental influence. These findings are consistent with previous studies that demonstrated heritability of narcissism but little shared environmental impact on narcissism [6]–[10]. In line with *hypothesis* 2, we have identified that intrapersonal grandiosity and interpersonal entitlement have different genetic and environmental bases. About 92–93% of their genetic and environmental contributions can be explained by unique genetic and unique environmental factors, respectively. Common genes and environments, correspondingly, accounted for only 7–8% of the genetic and environmental influences.

The findings have important implications. First, these results deepen our understanding of the heritability of narcissism: not only global narcissism is heritable [6]–[10], but its dimensions, in particular, intrapersonal grandiosity and interpersonal entitlement, are also heritable, suggesting neither grandiosity nor entitlement is trivial according to the first law of behavioral genetics [39]. Second, consistent with behavioral evidences [24]–[28], our twin study shows that the differential nature of intrapersonal grandiosity and interpersonal entitlement has genetic and environmental bases and the difference between them is fundamental but not trivial, providing novel evidence for the rationale for distinguishing between intrapersonal and interpersonal dimensions of narcissism. Third, our results echo with neuroimaging findings of different neural substrates for biased self-positivity [40]–[43], which encompasses grandiosity [19], [24], and empathy [44], [45], a characteristic closely related to entitlement [29]. It is likely that distinct genetic and environmental factors modulate grandiosity and entitlement via such different brain regions (e.g., media orbitofrontal cortex, right anterior insula). Finally, our findings also suggest that the intrapersonal and interpersonal distinction, which is very common in personality psychology [46]–[48], may also have genetic basis. More neuroimaging and twin studies, of course, are needed.

The limitations to the significance of our study are notable. First, both the intrapersonal and interpersonal dimensions of narcissism include multiple characteristics. We only measured one representative component for each of them, that is, grandiosity and entitlement, respectively, leaving other components, such as lack of empathy for interpersonal dimension and exhibition for intrapersonal dimension, unexamined. Future twin studies may examine other components or use more comprehensive measures of these two dimensions. A second limitation is our relatively small sample, which does not allow us to examine possible gender differences in genetic and environmental effects, to identify potential shared environmental influence such as culture, and to test whether genes interact with environments in their influences on narcissism. Twin studies with larger sample and more sophisticated design are definitely desirable to address these issues.
